# Tuberculosis imitating malignancy: Unusual presentation as a metastatic tumor – A case report

**DOI:** 10.5339/qmj.2024.qitc.5

**Published:** 2024-04-01

**Authors:** Abdullah Mohammad Arshad, Theeb Sulaiman, Hosamadean Maawia Benghashir, Husam Nabil Aldubai, Mousa Shaher Hussein

**Affiliations:** 1 Pulmonology Department, Hamad Medical Corporation, Doha, Qatar Email: aarshad1@hamad.qa; 2 Pathology Department, Hamad Medical Corporation, Doha, Qatar; 3 Infectious Disease Department, Hamad Medical Corporation, Doha, Qatar; 4 Department of Clinical Medicine, Qatar University, Doha, Qatar

**Keywords:** Tuberculosis, metastatic, malignancy, vocal cord, necrotizing

## Introduction

Tuberculosis (TB), caused by Mycobacterium tuberculosis, remains a significant global health challenge. Its classic pulmonary presentation includes cough, fever, weight loss, and hemoptysis. However, TB can manifest atypically and keep surprising clinicians, leading to diagnostic delays.^1,2^ Here we present a case report describing a unique presentation of TB that initially mimics malignancy with hoarseness of voice, a lung mass, and liver lesions.

## Case Presentation

A 44-year-old previously healthy gentleman was referred to the pulmonary clinic from the ENT clinic due to hoarseness of voice secondary to vocal cord palsy and abnormal chest X-ray. He had been complaining of hoarseness of voice for 8 months and minimal dry cough for 2 months. He denied shortness of breath, fever, weight loss, or night sweats. Physical examination revealed decreased breath sounds in the left upper lung field. Routine blood investigations were within normal limits.

**Figure 1.**
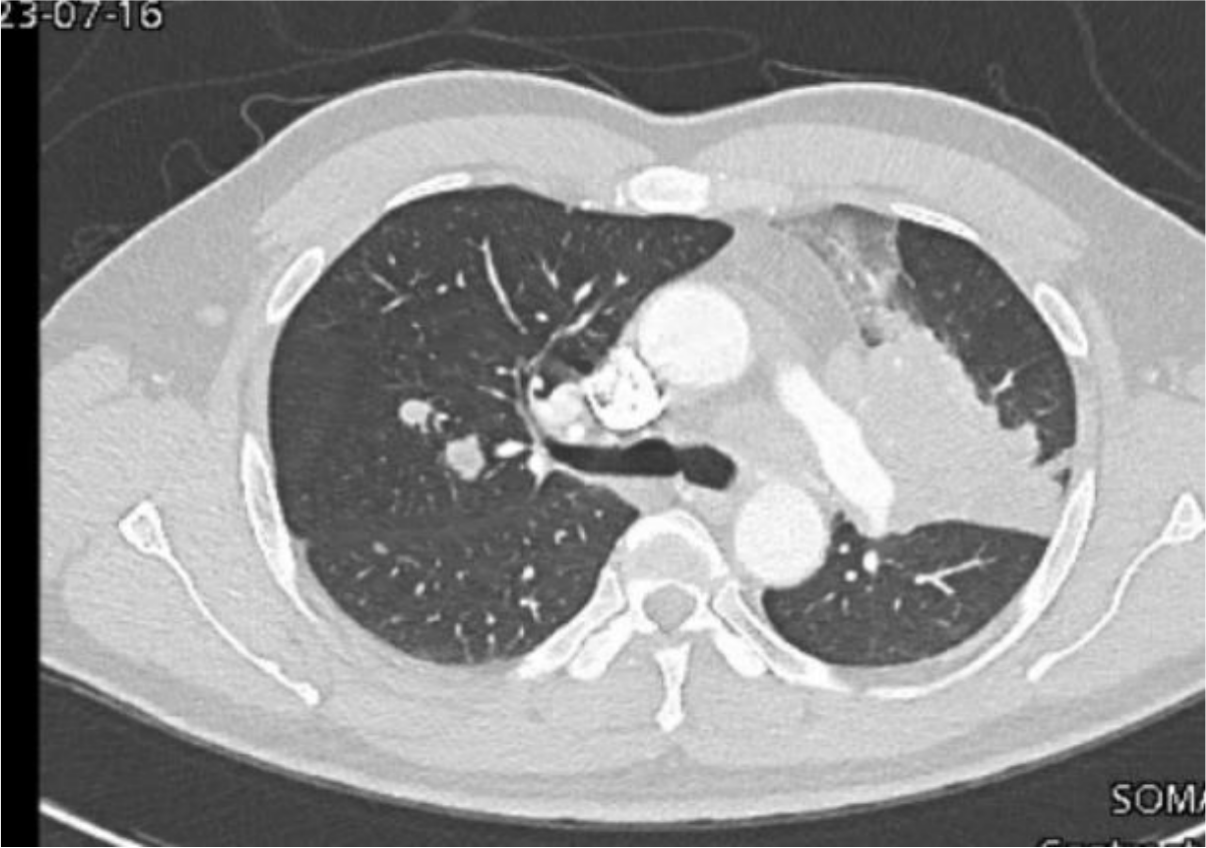
Large left lung mass with multiple bilateral pulmonary nodules.

**Figure 2.**
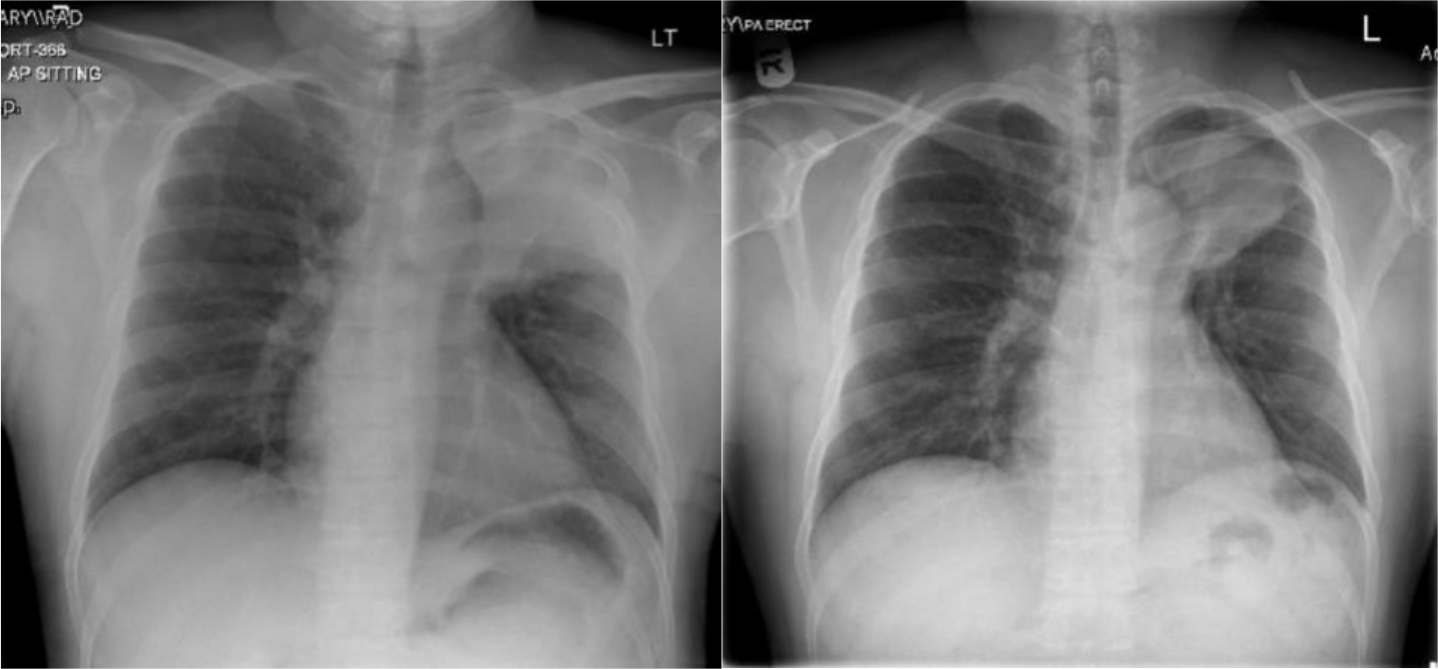
(a) homogenous opacity in the left upper lobe, (b) partial resolution after 3 months of treatment.

The chest X-ray revealed left-sided homogenous opacity, which required a contrast-enhanced computed tomography (CT) scan of the chest and abdomen. This revealed a large mass in the left upper lobe encasing the left hilar structures, with multiple bilateral nodules along with enlarged mediastinal lymph nodes and multiple lesions in the liver concerning for metastatic malignancy (Figures 1 and 2a).

**Figure 3.**
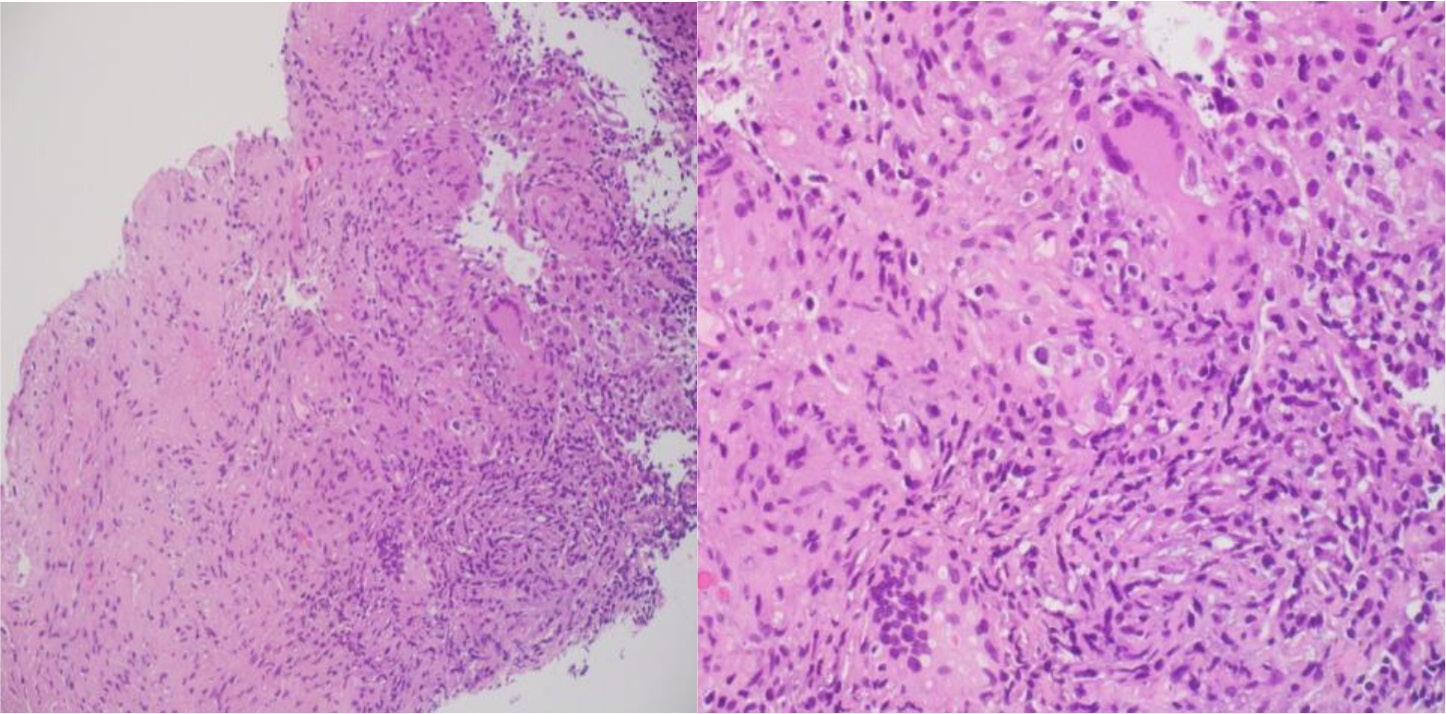
Lung mass biopsy showing necrotizing granulomatous inflammation.

Based on the clinical presentation and the radiological findings, the initial suspicion was lung cancer with metastasis to the liver. However, a CT-guided biopsy of the lung followed by liver lesion showed necrotizing granulomatous inflammation and positive TB PCR, confirming the diagnosis of TB (Figure 3). He was started on anti-tuberculous therapy and the follow-up showed improvement clinically and radiologically (Figure 2b).

## Conclusions

This case emphasizes the need for clinicians to maintain a high index of suspicion for TB, even when clinical and radiological findings initially suggest malignancy. Prompt diagnosis and appropriate treatment can lead to favorable outcomes, as demonstrated in this case.

## Conflict of Interest

There is no conflict of interest in regards to this abstract.
